# Suppressing the NHEJ pathway by DNA-PKcs inhibitor NU7026 prevents degradation of HBV cccDNA cleaved by CRISPR/Cas9

**DOI:** 10.1038/s41598-019-38526-6

**Published:** 2019-02-12

**Authors:** Dmitry Kostyushev, Anastasiya Kostyusheva, Sergey Brezgin, Dmitry Zarifyan, Anastasiya Utkina, Irina Goptar, Vladimir Chulanov

**Affiliations:** 1 Central Research Institute of Epidemiology, Viral Hepatitis, Moscow, 111123 Russian Federation; 2 Institute of Immunology, Federal Medical Biological Agency, Moscow, 115478 Russian Federation; 3 Izmerov Research Institute of Occupational Health, Gene Engineering and Biotechnology, Moscow, 105275 Russian Federation; 4 I.M. Sechenov First Moscow State Medical University, Infectious Diseases, Moscow, 119146 Russian Federation

## Abstract

Chronic hepatitis B is a severe liver disease caused by hepatitis B virus (HBV) infection. Covalently closed circular DNA (cccDNA), a super-spiralized, double-stranded form of the HBV genome, is the major determinant of viral persistence. CRISPR/Cas9 nucleases have been recently shown to introduce double-stranded DNA breaks into HBV cccDNA. The inflicted damage results predominantly in erroneous repair of cccDNA by non-homologous end-joining (NHEJ). NHEJ has been suggested to enhance anti-HBV activity of CRISPR/Cas9 and increase cccDNA mutation. In this study, we assessed anti-HBV activity of CRISPR/Cas9 and cccDNA repair outcomes in an altered NHEJ/HR environment. NU7026, a strong inhibitor of NHEJ, prevented CRISPR/Cas9-mediated degradation of cccDNA and resulted in frequent on-target deletions. We conclude that CRISPR/Cas9 is a highly effective tool to degrade cccDNA and first demonstrate that inhibiting NHEJ impairs cccDNA degradation.

## Introduction

Hepatitis B virus (HBV) chronically infects 250 million people worldwide, and more than a million people die from consequences of chronic hepatitis B (CHB), mainly cirrhosis and hepatocellular carcinoma (HCC)^1–3^. HBV belongs to the family *Hepadnaviridae*. The viral genome of the enveloped HBV particle comprises a circular, partially double-stranded DNA that is synthesized from an RNA intermediate using a reverse transcriptase. Upon entry, HBV virions are uncoated, and relaxed circular DNA is transported into the nucleus where it is repaired to form covalently closed circular DNA (cccDNA). cccDNA is the key component of that HBV life cycle that serves as a template for transcription of all viral mRNAs including pre-genomic RNA (pgRNA)^4–6^.

Currently approved therapies suppress HBV replication, but do not target cccDNA, which persists in the nuclei of infected hepatocytes^7^. A sterilizing cure of HBV infection requires eliminating cccDNA^8^. The clustered regularly interspaced short palindromic repeats/Cas9 nuclease (CRISPR/Cas9) system is a novel genome-editing tool that requires only two components to promote genome editing: (1) a Cas9 endonuclease, and (2) a short guide RNA (sgRNA) that targets the Cas9 protein to a specific site in the DNA. Bacterial systems of CRISPR/Cas9 nucleases are now frequently used to target HBV cccDNA in infected cells. HBV DNA is cut by a Cas9 nuclease recruited to the site of interest in the HBV genome by sgRNA^9^.

Although mammalian cells have complex mechanisms to repair damaged DNA, blunt-ended DNA double-stranded breaks (DSBs) are assumed to be degraded or repaired by two major competing pathways: the error-prone canonical non-homologous end-joining (NHEJ), or error-free homologous recombination (HR)^10^. NHEJ is the predominant form of DSB repair in mammalian cells, operating in all phases of the cell cycle and comprising two pathways: canonical and non-canonical (alternative)^11^. On the other hand, HR can only occur during the late S and G2 phases^12^. In canonical NHEJ, induction of DNA DSBs leads to the recruitment of KU70/80 heterodimer to the ends of damaged DNA, followed by interaction with the catalytic subunit of DNA-dependent protein kinase (DNA-PKcs), an important serine/threonine kinase, at the two broken DNA ends to generate the DNA-PK holoenzyme. DNA-PK acts as a scaffold protein that tethers the broken ends together. A cascade of protein phosphorylation reactions ultimately results in the excision of single-stranded overhangs of broken ends and ligation of DNA ends by the complex of ligase IV, XRCC4, and XLF^13^. The HR pathway requires a homologous DNA template to form an intact, proof-read DNA. HR is more intricately regulated: MRE11-RAD50-NGS1 complex first recognizes the DSBs and trims DNA ends to form 3′-overhangs of single-stranded DNA (ssDNA), while RPA stabilizes the ssDNA ends to prevent formation of secondary structures and is then substituted by RAD51, a key component of HR that coats ssDNA to form nucleoprotein filaments essential for the homology search and strand exchange. cccDNA DSB repair by c-NHEJ is error-prone, resulting in generation of short insertions or deletions (indels) and in-frame or frameshift short nucleotide polymorphisms (SNPs). Alternatively, mutation rates in HR are extremely low^14^. It is widely accepted that NHEJ and HR pathways are complementary^15^, with inhibition or deficiency of NHEJ components significantly elevating HR efficiency and *vice versa*. In certain circumstances, other back-up pathways, such as microhomology-mediated end-joining (MMEJ)/non-canonical NHEJ or single-strand annealing (SSA) may be implicated in DSB repair^16^. MMEJ commonly entails error-prone repair independently of DNA-PK, causing variable-size deletions and inserted nucleotides, whereas SSA generates large deletions^17,18^.

Upon HBV cccDNA cleavage using a single sgRNA, CRISPR/Cas9 systems induce indels in the targeted site^19,20^, while multiplex CRISPR/Cas9 targeting with several HBV-specific sgRNAs results primarily in degradation of HBV cccDNA^21^. Although CRISPR/Cas9 are very robust and straightforward gene editing tools, HBV cccDNA targeting with one CRISPR/Cas9 system per infected cell only modestly decreases the cccDNA pool, with over 30% of total cccDNA remaining in the cells 3 weeks after CRISPR/Cas9 transduction, and 10% 5 weeks after^22^. In an elegant study by Seeger *et al*.^23^, infecting HepG2-NTCP cells producing CRISPR/Cas9 with HBV particles resulted in very frequent on-target mutations (SNPs and small indels), and only a small portion of the HBV genomes retained its wild-type genome sequence^23^. It should be noted that HBV genomes with on-target mutations corresponding to the sgRNAs sequence will mostly escape CRISPR/Cas9 activity and will not be cleaved repetitively due to limited mismatch tolerance of CRISPR/Cas9^24^. Single mismatches in the target region, nucleotide indels, or fewer than 6 mismatches in distal regions of sgRNA are well tolerated by most type II CRISPR/Cas9 systems^25,26^. Thus, mutated HBV cccDNA (either replication-competent or not) can remain in cells after nucleolytic cleavage. However, that study uses an artificial situation in which the cells to be infected already express an anti-HBV CRISPR/Cas9 system. In contrast, many studies in which infected cells with a well-established HBV life cycle were transduced with anti-HBV CRISPR/Cas9, the majority of HBV cccDNA genomes remained intact^21^. Given the very high nucleolytic activity of CRISPR/Cas9 towards incoming HBV genomes, the reasons for such discrepancy in cccDNA cleavage could be that a portion of cccDNA is not accessible to Cas9 proteins (e.g., epigenetically silenced cccDNA), or that cccDNA is cleaved by CRISPR/Cas9 but is repaired by HR without on-target mutagenesis.

The reliance of cccDNA repair on NHEJ and HR prompted us to modulate activity of DSB repair pathways to alter outcomes of cccDNA nucleolytic cleavage. We hypothesized that pharmacologically inhibiting NHEJ concurrently with using an HBV-targeting CRISPR/Cas9 system would overwhelm DNA repair systems and result in degradation of cccDNA, while suppression of HR would potentiate NHEJ and increase cccDNA mutation rates. Previous screens of thousands of chemical compounds identified pharmacological inhibitors and activators of NHEJ and HR^27^. In particular, L755507 (L755), a potent β3-adrenergic receptor partial agonist, was shown to enhance HR repair to improve genome editing. Another molecule, 3′-azido-3′-deoxythymidine (3-aza), a reverse transcriptase inhibitor previously used in HIV-1 therapy, enhances NHEJ activity. Moreover, inhibition of DNA-PKcs by small molecule inhibitors NU7441 and KU-0060648 drastically reduced the frequency of NHEJ while increasing the rates of HR^28^. Similarly, degradation of ligase IV by adenoviral protein E1B (Ad4E1B) was shown to considerably increase HR rates at the expense of NHEJ^29^. To block NHEJ in this study, we used NU7026, a potent DNA-PK inhibitor, reported to have 60-fold higher activity against DNA-PK than PI3K, which has a very similar domain structure of the catalytic domain, and is not active against ATM and ATR kinases^30^. We inhibited HR by B02, a small molecule inhibitor that disrupts RAD51 foci formation^31^. We analyzed anti-HBV activity and cccDNA cleavage outcomes of CRISPR/Cas9 targeting and enhancing NHEJ (with 3-aza) and HR (with L755) or suppressing NHEJ and HR using NU7026, Ad4E1B, or B02.

## Results

### Toxicity of small molecules and effects on viability and cell cycle

**Figure 1:**
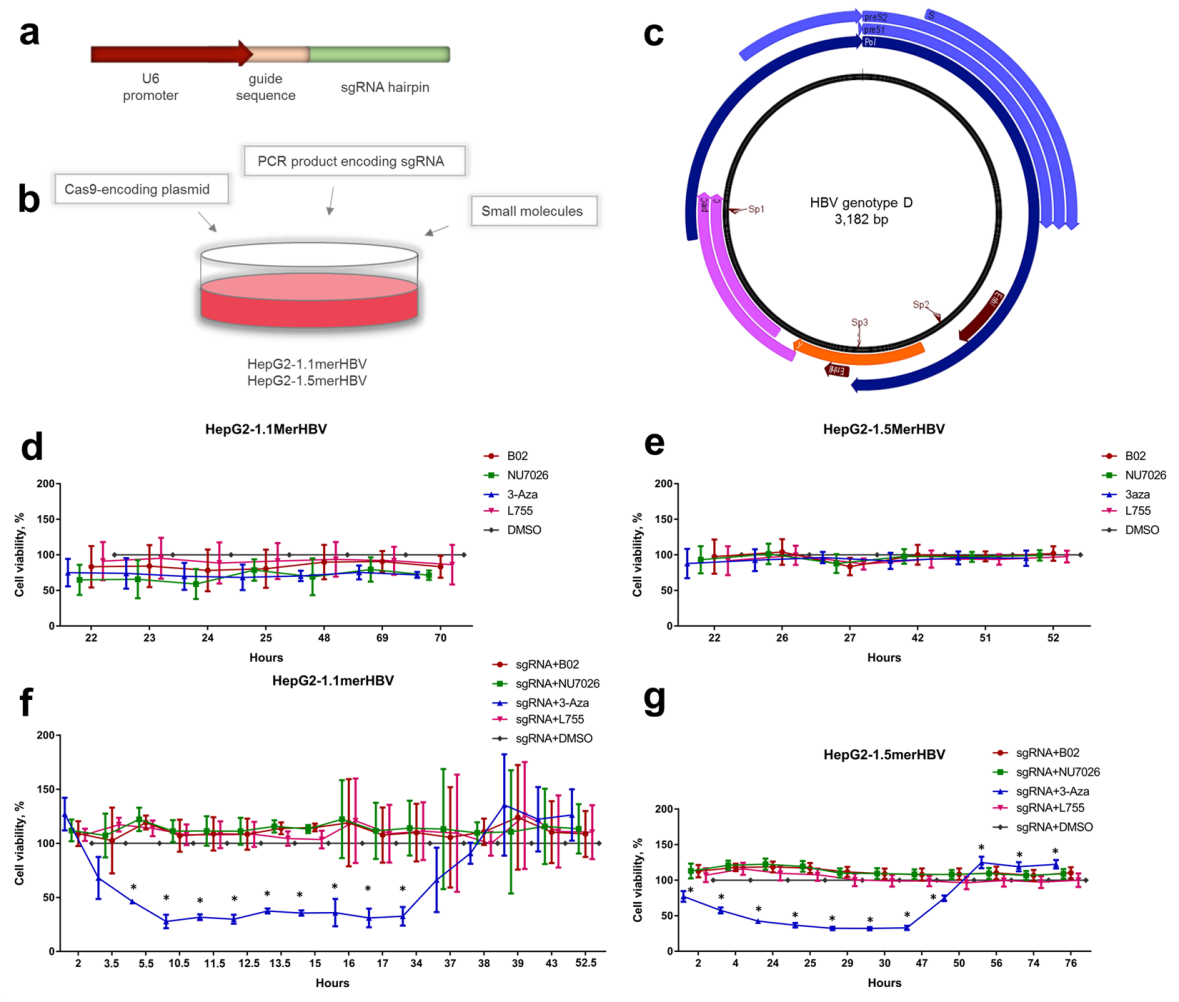
Experimental design and cytotoxicity studies. (**a**) Structure of PCR products encoding sgRNAs. (**b**) Experimental design. HepG2-1.1merHBV or HepG2-1.5merHBV cells were lipofected with a Cas9-encoding plasmid and a PCR product encoding an sgRNA, and were treated with the small molecules B02, NU7026, 3-aza, L755, or DMSO simultaneously. Untransfected cells were negatively selected using blasticidin. (**c**) SgRNAs (Sp1-Sp3) used in this study, targeting functional regions of the HBV genome. Effects of small molecules on cell viability in (**d**) HepG2-1.1merHBV or (**e**) HepG2-1.5merHBV cell lines treated with small molecules for 72 h and observed for the designated period of time. Effects of small molecules on (**f**) HepG2-1.1merHBV cells or (**g**) HepG2-1.5merHBV cells 3 d post transfection with HBV-targeting CRISPR/Cas9 and concomitant treatment with small molecules. Asterisks indicate statistically significant differences. *p < 0.05.

Prior to analyzing the effects of the small molecules on CRISPR/Cas9-mediated anti-HBV activity, we determined their potential cytotoxic and genotoxic effects. Compound concentrations and treatment duration were based on those previously shown to be non-toxic in HepG2 and other cell lines, but effective for modulating NHEJ/HR pathways^27,32–35^. HepG2-1.1mer and HepG2-1.5merHBV cells were seeded onto 96-well plates at 30% confluency, transfected or not with HBV-targeting CRISPR/Cas9, and treated with NHEJ/HR enhancers or inhibitors (Fig. 1a–c). Control cells were treated with DMSO. At the selected concentrations, none of the compounds affected cell viability when used alone (Fig. 1d,e). However, upon transfection of CRISPR/Cas9, 3-aza decreased viability of HepG2-1.1mer and HepG2-1.5mer cells by 2-fold within the first 34 h and 50 h, respectively (Fig. 1f,g). As toxicity of 3-aza and L755 have been exhaustively investigated in previous experiments, we focused on B02 and NU7026, potent inhibitors of HR and NHEJ, respectively. As expected, flow cytometry analysis of cell cycle distribution demonstrated that the majority of cells (>79% in all groups) were arrested in G1/G0 phase (Fig. S1a). Indeed, HBV was previously shown to dysregulate the G1 to S transition^36^. Incubation with NU7026 and B02 mildly increased the proportion of cells in G1/G0, while treatment with either NU7026 or B02 decreased the proportions of cells in S-phase and G2/M compared to controls (Fig. S1a). Analysis of apoptosis using morphological assessment of cell nuclei (see Materials and Methods) did not reveal significant differences between experimental groups and DMSO-treated control (Fig. S1b). Genotoxicity of NU7026 and B02 was assessed by immunostaining and counting foci of yH2AX, Ser-139 phosphorylated form of H2AX protein, a reliable marker of DNA damage response in the cells^37^. Few yH2AX foci (0.09 ± 0.11) were detected in control cells (Fig. SS1c,d), but incubation with B02 resulted in generation of small yH2AX foci (2.78 ± 0.98 per cell). NU7026 did not induce yH2AX foci formation. Incubation with H_2_O_2_ for 1 h served as a positive control, inducing generation of numerous yH2AX puncta.

### Design of CRISPR/Cas9

To determine effects of the small molecules on anti-HBV activity of CRISPR/Cas9, we lipofected HepG2-1.1merHBV cells with SpCas9-encoding vector and PCR products (Fig. 1a,b) encoding one of the highly effective sgRNAs identified previously (unpublished data). We utilized 3 sgRNAs (Fig. 1c; Table S1-3) targeting core/pre-core (Sp1), EnhI (Sp2), and X-gene (Sp3) regions. On the day of transfection, cells were treated with small molecules, which were not removed until the end of the experiment. Transfection of HepG2 cell lines was highly efficient (>80% transfected cells; data not shown), and untransfected cells were removed by negative selection with blasticidin.

### Anti-HBV activity of CRISPR/Cas9 in an altered NHEJ/HR environment

First, we analyzed the effects of the small molecules on the HBV life cycle *per se* in HepG2-1.1merHBV and HepG2-1.5merHBVcell lines. In HepG2-1.1mer cells, pgRNA is expressed via a Tet-on inducible cytomegaloviral promoter, so HBV replicates and cccDNA is formed *de novo* only after doxycycline is added to the culture medium. In HepG2-1.5merHBV cells, HBV is produced constitutively under a wild-type promoter.

HBV pgRNA and S-RNA levels were down-regulated in both cell lines upon treatment with L755 or B02 (Fig. S2a,c), while HBV DNA and cccDNA levels were not greatly affected by either L755 or B02 (Fig. S2b,d). Incubation with 3-aza down-regulated levels of almost all HBV intermediates measured, including HBV DNA and cccDNA (Fig. S2a–d), which might be attributed to its general toxicity (Fig. 1f,g). Inhibiting NHEJ by Ad4E1B had no effect on the HBV life cycle (Fig. S2a,b). In contrast, inhibiting NHEJ by treatment with the DNA-PKcs inhibitor NU026 suppressed HBV transcription more than 2-fold (Fig. S2a). Generation of cccDNA *de novo* and intracellular levels of HBV DNA were statistically significantly reduced by NU7026 as well (Fig. S2b), but these results were not reproduced in HepG2-1.5merHBV cell line. Thus, treatment with certain NHEJ/HR enhancers and inhibitors perturbs the HBV life cycle, but effects of small molecules are not very profound and vary between two cell lines.

**Figure 2:**
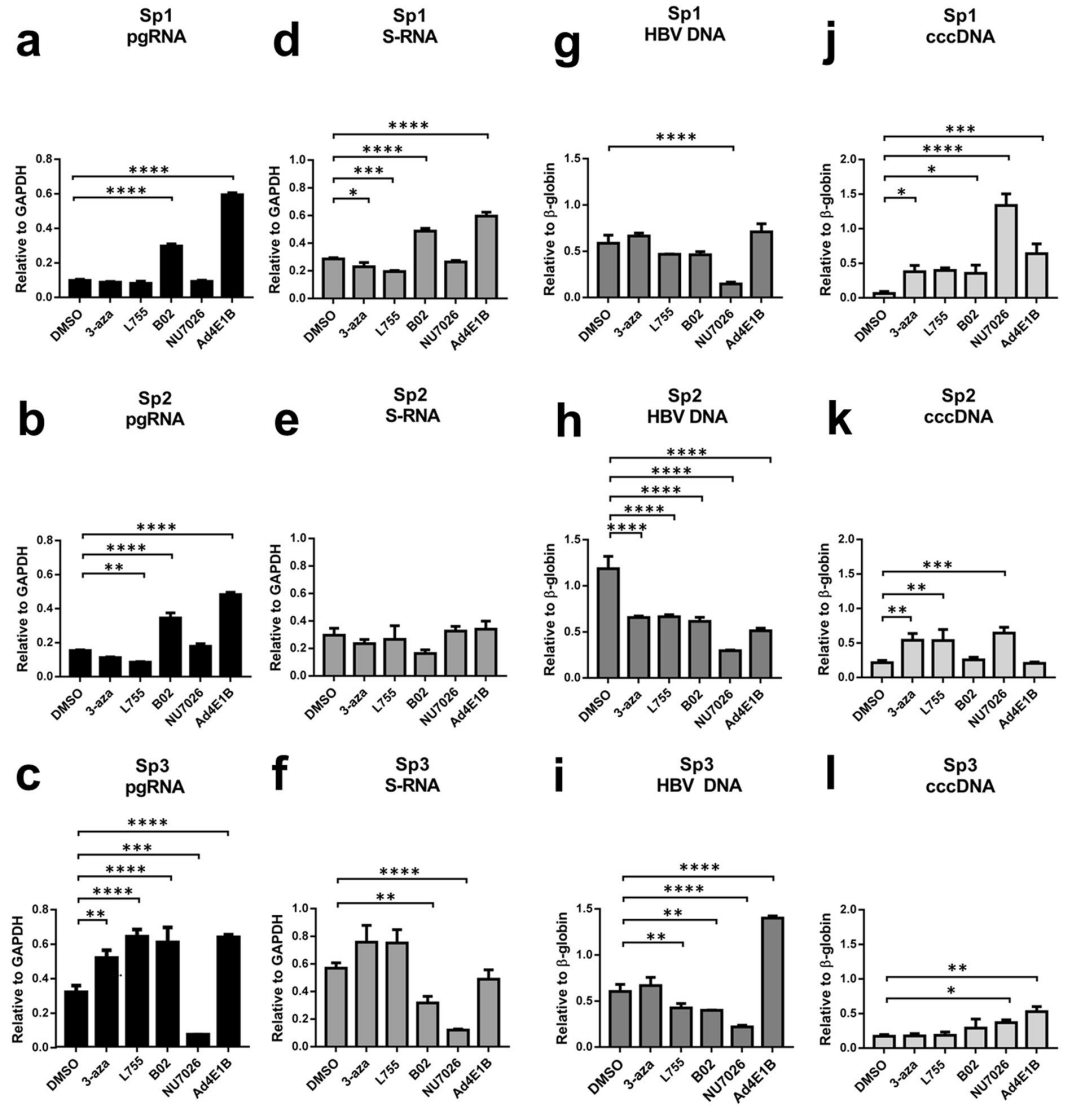
Effects of small molecules 3-aza, L755, B02, and NU7026, and the protein Ad4E1B on HBV replication and CRISPR/Cas9-mediated suppression of HBV in HepG2-1.1merHBV cells. (**a**–**c**) Relative levels of pgRNA, (**d**–**f**) S-RNA, (**g**–**i**) intracellular HBV DNA, and (J-L) cccDNA in HepG2-1.1merHBV cells transfected with CRISPR/Cas9 system and one of the 3 sgRNA (Sp1, Sp2, Sp3), as indicated. Asterisks indicate statistically significant differences. *p < 0.05, **p < 0.01, ***p < 0.001, ****p < 0.0001.

Transfection of HBV-targeting CRISPR/Cas9 systems into HepG2-1.1merHBV cells resulted in strong suppression of HBV transcription (Fig. 2). Similar to anti-HBV activity in HepG2 co-transfection experiment, all parameters of HBV life cycle were significantly repressed (unpublished results). pgRNA levels dropped by over 60–70% in each DMSO group (Fig. 2a–c). Importantly, a 2-fold decline in S-RNA levels was observed (Fig. 2d–f). Along with strongly suppressing HBV transcription, transfection of CRISPR/Cas9 significantly reduced intracellular HBV DNA and cccDNA levels (Fig. 2g**–**l). Thus, the CRISPR/Cas9 systems were very effective in cleaving episomal HBV cccDNA and the integrated HBV genome, which transcribes S-RNA independently of the Tet-on system.

**Figure 3:**
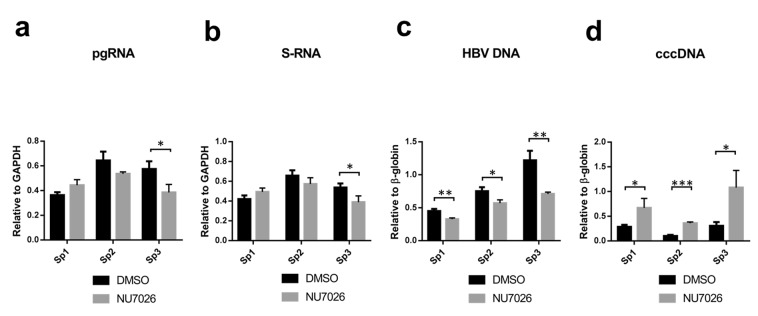
Effects of NU7026 on anti-HBV activity of CRISPR/Cas9 in HepG2-1.5merHBV cells. (**a**–**d**) Differences in the levels of indicated HBV intermediates after transfection with CRISPR/Cas9 and treatment with either DMSO or NU7026. HBV pgRNA and S-RNA levels were measured relative to GAPDH RNA levels; HBV DNA and cccDNA were measured relative to β-globin levels.

Treatment of transfected cells with 3-aza or L755 consistently enhanced or slightly inhibited CRISPR/Cas9-mediated anti-HBV activity, respectively. Inhibiting HR by B02 had no consistent effect on antiviral activity of CRISPR/Cas9. Co-expression of Ad4E1B, a factor inhibiting NHEJ, resulted in lower HBV suppression than Cas9 alone (Fig. 2). In contrast, when NU7026 was added to transfected cells, intracellular HBV DNA levels dropped much lower with every sgRNA used compared to DMSO-treated group, resulting in 2.85–3.97-fold increase in antiviral efficacy (Fig. 2J–L). Effects on transcription were similar between DMSO-treated and NU7026-treated groups (Fig. 2a–f). However, relative levels of HBV cccDNA either remained at the level of mock control when using Sp1 sgRNA or were significantly higher compared to DMSO-treated group (Sp2 and Sp3) (Fig. 2j**–**l). Similarly to HepG2-1.1mer cells, NU7026 treatment of CRISPR/Cas9-transfected HepG2-1.5merHBV cells prevented HBV cccDNA degradation by CRISPR/Cas9 (Fig. 3d), whereas HBV DNA levels were consistently lower upon treatment with NU7026 (Fig. 3c). HBV pgRNA and S-RNA levels were not significantly affected by NU7026 (Fig. 3a,b).

Thus, CRISPR/Cas9 systems were very effective in cleaving HBV cccDNA and integrated HBV DNA, as indicated by all parameters tested. Among all NHEJ/HR inhibitors and enhancers tested, only NU7026 significantly affected CRISPR/Cas9-mediated anti-HBV activity.

### Inhibition of NHEJ by NU7026 results in CRISPR/Cas9-mediated hyper-editing of HBV cccDNA

**Figure 4:**
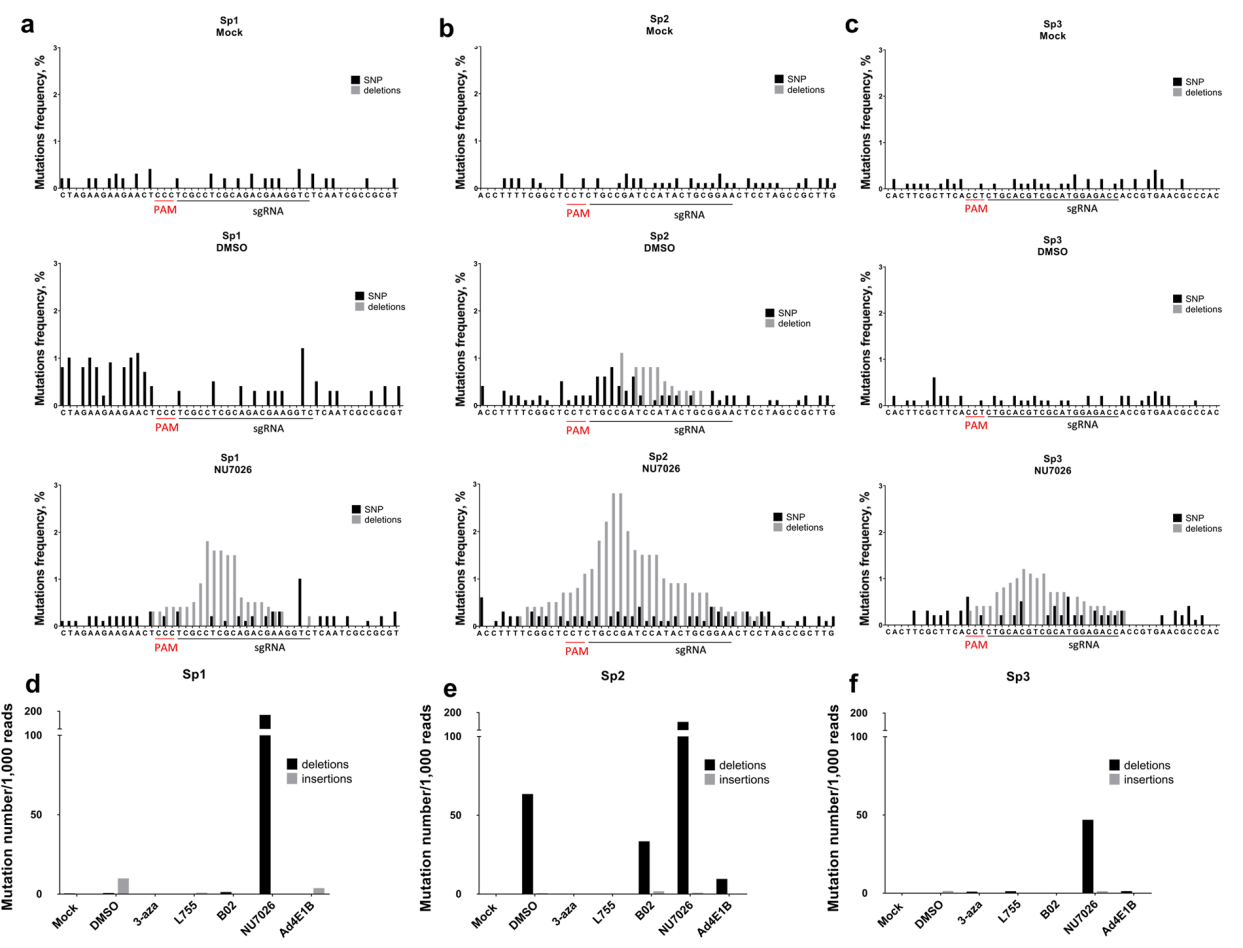
Deep sequencing of CRISPR/Cas9 on-target sites. (**a**–**c**) Mutation rates and profiles of DSB repair outcomes in the target regions of HBV cccDNA isolated from HepG2-1.1merHBV cells. Each sgRNA (Sp1, Sp2, Sp3) has a mock control group, DMSO control group, and groups treated with NU7026 (see Supplementary Fig. S4 for results of treatment with other small molecules). Mock group was transfected with a Cas9 vector and non-targeting PCR product and treated with DMSO. Control groups were transfected with a Cas9 vector and an HBV-targeting sgRNA encoded by a PCR product, and treated with DMSO. PAM sequence for all sgRNAs was NGG (the strand complementary to the target DNA is provided). Regions containing the on-target sites were sequenced, and frequencies of insertions/deletions were calculated for sgRNAs Sp1 (**d**), Sp2 (**e**), and Sp3 (**f**) in cells treated with 3-aza, L755, B02, and Ad4E1B compared to mock control group and to DMSO control group. Number of indels per 1000 reads was counted for all experimental groups with corresponding sgRNAs.

We amplified and deep-sequenced short regions of HBV cccDNA overlapping the CRISPR/Cas9 target sites to analyze alterations in DSB repair outcomes. In our experimental setting, mutations upstream of PAM were detected only in the Sp2 transfection group (Fig. 4b); the mutation rate was not different from mock-treated control in the other groups (Figs 4, S3). These results are inconsistent with the observed decrease in HBV intermediates and transcription levels, and might be attributable to preferential degradation of cleaved cccDNA, as observed earlier. Surprisingly, among all small molecules tested, only NU7026 profoundly affected indel formation (Figs 4 and S3,S4). Treatment with NU7026 resulted in numerous deletions with a typical distribution pattern around the preferential cut site at nucleotide 3–6 upstream of PAM.

**Figure 5:**
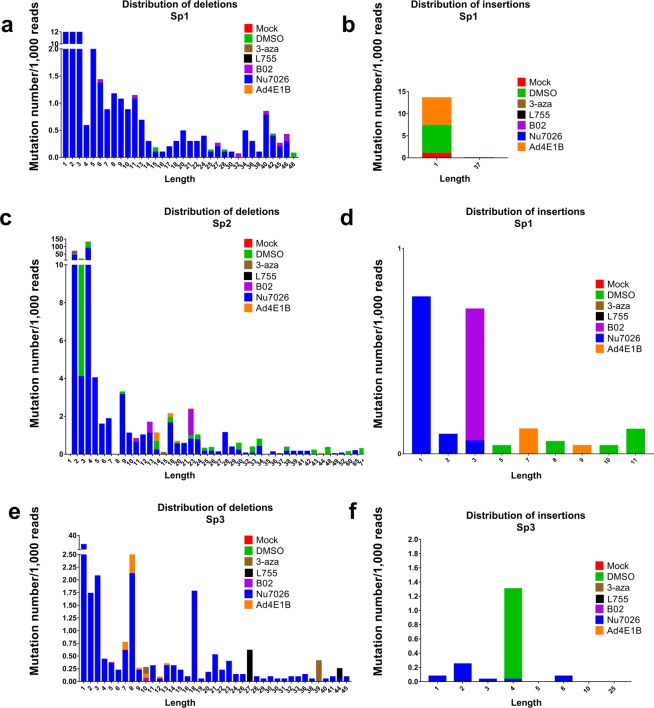
Distribution of insertions/deletions by length. Sequencing data from all experimental groups were analyzed to provide distribution of deletions (**a**,**c**,**e**) and insertions (**b**,**d**,**f**) induced by CRISPR/Cas9 at the sites targeted in the HBV genome by sgRNAs Sp1, Sp2, or Sp3.

Compared to DMSO control groups, NU7026 enhanced deletion formation upon CRISPR/Cas9 transfection, with deletion frequency as high as 180–200 per 1,000 reads (Fig. 4d–f). NU7026 did not result in as many insertions as deletions (Figs 4d–f and 5b,d,f). Analysis of deletion length demonstrated that NU7026 induced frequent, short deletions and deletions up to 60 nucleotides in length (Fig. 5a,c,e).

Our findings reinforce the previous data that CRISPR/Cas9 systems are very effective in cleaving HBV cccDNA, at least *in vitro*. Modulating NHEJ/HR pathways by previously identified CRISPR/Cas9 small molecule enhancers does not significantly contribute to the antiviral efficacy of CRISPR/Cas9. In sharp contrast, treating cells with NU7026, a potent and specific inhibitor of DNA-PKcs, profoundly affected cccDNA DSB repair outcomes, prevented degradation of nucleolytically cleaved cccDNA, and may have increased anti-HBV activity of site-specific nucleases.

## Discussion

Recent breakthroughs in gene editing technology have enabled highly specific and effective cleavage of target viral DNA^38^. HBV is an ideal target for CRISPR/Cas9 nucleases, as cccDNA, the major form of the viral genome responsible for chronic hepatitis B, is well-conserved, accessible to nucleases, and double-stranded. Thus, it is possible to design universal sgRNAs targeting all HBV genotypes to cut cccDNA at specific loci and dampen viral replication. However, a proportion of HBV cccDNA is not degraded upon CRISPR/Cas9-mediated cleavage^23^. The DSBs induced by CRISPR/Cas9 are sealed by NHEJ pathway, commonly resulting in indels or SNP mutations. Ramanan *et al*.^22^ demonstrated that cccDNA levels decrease >95% after 4 weeks of lentiviral transduction of CRISPR/Cas9 in cell culture^22^. Kennedy *et al*.^39^ achieved very effective suppression of HBV replication 2 weeks post lentiviral transduction of CRISPR/Cas9^39^, so that the levels of cccDNA were significantly reduced, and the remaining cccDNA contained frequent indel mutations. It should be noted that in previous experiments^23,39^ targeting HBV by CRISPR/Cas9, higher rates of cccDNA mutations were observed, and reductions in cccDNA levels were not as rapid and significant as in our hands. This may be explained by different methodology, in particular, the key difference is that previous experiments relied on lentiviral transduction of CRISPR/Cas9 into the cells. Here, we immediately transfected large quantities of CRISPR/Cas9 into the cells, and this led to preferential degradation of HBV cccDNA (Figs 2j–l, 3d and 4). Several recent studies demonstrated that higher Cas9/sgRNA threshold expression results in more efficient genome editing^40,41^. We suggest that HBV genome is preferentially degraded after generation of a DNA DSB when CRISPR/Cas9 components are overabundant.

Approaches for enhancing CRISPR/Cas9 activity are being actively investigated, and include Cas9 protein modifications^42^, sgRNA scaffolds^43^, Cas9 proteins with new properties^44^, and small molecules to modulate DNA DSB repair outcomes^28^. In several reports, NHEJ was hypothesized to enhance the antiviral activity of site-specific nucleases^27,45^. Modulating DNA damage response factors was proposed as a tool to determine the fate of cleaved cccDNA and as a potential antiviral strategy^23^. Given the high versatility of the currently available tools to temporarily modulate activity of DNA damage response factors, this appeared to be a plausible approach. Thus, we decided to directly assess how NHEJ/HR pathways are implicated in anti-HBV activity of CRISPR/Cas9 and the fate of the cleaved cccDNA.

Unbiased screening of thousands of small molecules for cancer research and, more recently, for the purposes of CRISPR/Cas9-mediated gene editing, identified chemical agents capable of specifically blocking NHEJ (NU7026, Ad4E1B) or HR (B02), or enhancing CRISPR/Cas9 activity (3-aza, L755)^27,45,46^. CRISPR/Cas9 was very effective against HBV in our hands. In particular, HBV pgRNA and cccDNA levels were reduced >80% (Figs 2 and 3). At the same time, small molecules 3-aza, L755, B02 and adenoviral protein Ad4E1B had mild or no effect on either anti-HBV activity or outcomes of CRISPR/Cas9-mediated cleavage (Fig. 2). Conflicting reports on the role of 3-aza and L755 in CRISPR/Cas9-mediated genome editing have been published recently^46^. It has been demonstrated that a number of small molecules, including L755, showed no consistent effect on CRISPR/Cas9 targeting, and can even have opposing effects of genome editing^46^. In our hands, neither of these small molecules affected CRISPR/Cas9 anti-HBV activity or their effects were very inconsistent (Figs 2 and S3). B02, inhibitor of RAD51 in the HR pathway, also had almost no effect either on anti-HBV activity or on DSB repair outcomes. Additionally, Ad4E1B, a protein that degrades DNA ligase IV in the NHEJ pathway, was proposed as a factor to inhibit NHEJ and refine gene editing techniques^29^. However, suppressing HBV replication was less significant when Ad4E1B was expressed concomitantly^29^. DSB repair outcomes were identical compared to mock control.

NGS sequencing of regions targeted by CRISPR/Cas9 detected rare mutations, indicating preferential degradation of HBV cccDNA in our experimental setting (Figs 2 and S3). In sharp contrast, while HBV cccDNA levels remained at the level of mock control when NU7026 was added to culture medium, sequencing revealed that a substantial portion of cccDNA contained numerous deletions of varying sizes (Fig. 4). Thus, cccDNA is subjected to highly effective nucleolytic cleavage, but inhibiting DNA-PKcs of the NHEJ pathway by NU7026 switches the fate of DSBs, and the damaged cccDNA may be exclusively repaired by the error-prone pathways instead of being degraded.

Currently, the mechanisms underlying the choice between the NHEJ or HR pathway are largely obscure^47,48^. A competition exists between NHEJ and HR pathways, depending on the enzymatic activity of certain factors, including DNA-PKcs^49^. However, alternative pathways may play important roles, with, for example, MMEJ or SSA repairing DSBs if the classic mechanisms are not functional^50^. It is not clear why inhibiting NHEJ by NU7026, which targets DNA-PKcs, a key enzyme downstream of the NHEJ pathway, followed by Cas9 cleavage generates cccDNA with numerous mutations, while inhibiting NHEJ by Ad4E1B, a protein targeting DNA ligase IV, does not. It is tempting to speculate that upon DNA-PKcs inhibition, cccDNA DSBs are preferentially repaired by alternative pathways. A recently characterized backup NHEJ pathway (alternative NHEJ) is activated when canonical NHEJ is suppressed^51^. It does not require NHEJ proteins and is highly error-prone^52^. Indeed, treating DNA-PKcs-deficient cells with etoposide, an agent that creates DSBs via DNA topoisomerase II poisoning) results in much higher rates of chromatid breaks and exchanges compared to etoposide treatment alone^53^.

In summary, our data challenge a previously suggested approach based on a combination of CRISPR/Cas9 systems and small molecules consigned to block NHEJ and enhance cccDNA degradation. Inhibiting NHEJ or HR mildly affected anti-HBV activity of CRISPR/Cas9, but inhibiting DNA-PKcs prevents degradation of nucleolytically cleaved cccDNA and may redirect cccDNA DSBs repair to alternative (backup) pathways. Recent discovery of large deletions in the human genome upon CRISPR/Cas9 cleavage may also be attributed to the malfunctioning of NHEJ/HR and operation of more erroneous backup DNA repair signaling^54^.

CRISPR/Cas9 systems are very effective against HBV cccDNA, and fine-tuning these systems, e.g., increasing Cas9/sgRNA threshold should improve antiviral activity and cccDNA decline. Classic CRISPR/Cas9 systems are notorious for their off-target activity^55^, especially when CRISPR/Cas9 components are vastly overexpressed in cells^56^, which might compromise their utility in a clinical setting. However, more precise CRISPR/Cas9 with more restrictive PAM hold a lot of promise for human gene therapy. Along with that, there is a lot of other aspects in CRISPR/Cas9 research that make translation of *in vitro* results into clinical practice very challenging, in particular, Streptococcus pyogenes Cas9-reactive T cells pre-exist in adult humans^57^, so that CRISPR/Cas9-based therapeutics may be less effective *in vivo*. Delivery tools based on viral vectors have numerous shortcomings, including potential immune responses, risks of carcinogenesis and limited insertion size^58^. Several groups used AAV-based vector systems^59,60^ and high-capacity adenoviral vectors^61^ for delivery of classic Streptococcus pyogenes Cas9 and smaller Cas9 orthologs. However, we consider targeting HBV *in vivo* using viral-based methods for long-term expression of CRISPR/Cas9 in humans impractical. According to our results, it is possible to eliminate the majority of HBV cccDNA *in vitro* within 6 days post transfection, while long-term production of CRISPR/Cas9 may have high a very risk of off-target activity, but it needs to be investigated in detail in further *in vivo* studies. Non-viral methods of CRISPR/Cas9 delivery are being actively developed and hold a lot of promise for developing CRISPR/Cas9-based anti-HBV therapeutics, they include gold nanoparticles^62,63^, exosome-liposome^64^, lipid nanoparticles^65^ etc.

We conclude that (a) the majority of HBV cccDNA is degraded in our setting. (b) Inhibiting DNA-PKcs prevents degradation of cccDNA, possibly by switching DSB repair to alternative pathways and resulting in a pool of cccDNA with numerous deletions. (c) Modulating NHEJ/HR by small molecules L755, 3-aza, and B02 and protein Ad4E1B does not significantly enhance antiviral properties of CRISPR/Cas9.

## Materials and Methods

### Cell culture and transfection

Human HepG2-1.1merHBV and HepG2-1.5merHBV cell lines were cultured in complete DMED high glucose medium with 10% FBS, 1% L-glutamine, and 1% penicillin/streptomycin. Lipofection of stable HepG2-1.1merHBV cells using Lipofectamine 3000 (Thermo Fisher Scientific) was performed according to manufacturer’s instructions. In brief, cells were seeded into 6-well plates 24 h before lipofection in complete medium without doxycycline (for HepG2-1.5merHBV) or with 100 ng/mL doxycycline (for HepG2-1.1merHBV). On the day of transfection, doxycycline-containing medium was discarded, cells were lipofected using Lipofectamine3000 with Lenti-Cas9-T2A-Blast plasmid and sgRNA-encoding PCR products in medium containing NHEJ/HR enhancers or inhibitors. Alternatively, pU6-(BbsI)_CBh-Cas9-T2A-BFP-P2A-Ad4E1B was co-transfected with blasticidin-encoding plasmid and PCR products. After 48 h, medium was discarded, cells were washed twice in PBS, and complete medium with small molecules was added for the next 24 h. After that, untransfected cells were removed by blasticidin (15 ng/mL) added for the following 120 h. All results were reproduced at least 3 times.

### Small molecules

Stocks of NU7026, B02, L755507, and 3-aza were frozen in DMSO and kept at −80 °C until use, when aliquots were thawed at room temperature and diluted in cell culture medium. Final concentrations of the small molecules were as follows: NU7026, 7.5 μM; B02, 3 μM; L755507, 5 μM; and 3-aza, 5 μM. Cells were incubated with small molecules for 72 h. Cells transfected with Cas9-encoding plasmids and PCR product and treated with DMSO were used as the negative control.

### Cell viability analysis

Cells were seeded at 30% confluency and treated with small molecules alone or after lipofection with plasmids encoding CRISPR/Cas9 systems. Cell viability was assessed using the Cell Cytotoxicity kit (Abcam) according to manufacturer’s instructions.

### CRISPR/Cas9 constructs

HBV genome targets were assessed and sgRNA designed using the open-access web tools Broad Institute Genetic Perturbation Platform and CCTop CRISPR/Cas9 target online calculator. PCR products with a U6 promoter and sgRNA sequence were synthesized by two-step mutagenic PCR using Q5 polymerase (primers are listed in Supplementary Table S1). PCR products were purified by Qiagen gel extraction kit. Concentration of synthesized PCR products was measured by Nanodrop2000. The following plasmids were used: Lenti-Cas9-2A-Blast (AddGene plasmid #73310) was a gift from Jason Moffat; pLX-sgRNA (AddGene plasmid #60662) was a gift from Eric Lander and David Sabatini; pU6-(BbsI)_CBh-Cas9-T2A-BFP-P2A-Ad4E1B (AddGene plasmid #64218) was a gift from Ralf Kuehn.

### Isolation of nucleic acids

Cell culture medium was discarded, and cells were washed twice with PBS before being lysed in AmpliSens Riboprep lysis buffer. Nucleic acids were isolated using the AmpliSens Riboprep kit (AmpliSens Biotechnologies) according to the manufacturer’s instructions. For RNA isolation, nucleic acids were treated with RNase-free DNase I (NEB) for 30 min at 37 °C, purified from the enzyme using AmpliSens Riboprep kit, and reverse transcribed using AmpliSens Reverta-FL. HBV cccDNA was isolated via HIRT procedure as described by Cai *et al*.^66^, followed by treatment with plasmid-safe ATP-dependent DNase (Epicentre) for 12 h at 37 °C and inactivating the enzyme at 72 °C for 15 min.

### PCR analysis

HBV pgRNA and S-RNA concentrations were measured relative to GAPDH mRNA as reference. Total intracellular HBV DNA and cccDNA levels were normalized to genomic β-globin. All PCRs were performed with specific sets of primers and probes (Supplementary Table S1). Relative expression levels were calculated via ddCt method.

### Immunofluorescence

Cells were seeded on glass coverslips, treated appropriately, and fixed in 4% paraformaldehyde for 10 min on the day of harvest, as previously described. Next, coverslips were washed 3 times in Tris-HCl (50 mM, pH 8.0), incubated for 30 min with blocking buffer (0.02% of Triton X-100, 10% horse serum, and 150 mM NaCl in Tris-HCl, 50 mM, pH 8.0), and incubated with primary rabbit anti-yH2AX antibodies (ab11175) at room temperature for 1 h. The cells were washed 3 times for 5 min in washing buffer (0.02% of Triton X-100 and 200 mM NaCl in Tris-HCl, 50 mM, pH 8.0), then incubated with secondary Alexa Fluor 488 goat anti-rabbit IgG antibodies (ab205718) with Hoechst33342 (1/10,000; ab228551) at room temperature for 1 h. Coverslips were washed 3 times for 5 min in washing buffer and mounted with Fluoroshield reagent (Abcam). Images were captured using a Leica DMI6000 microscope with 20× and 100× immersion objectives.

### Apoptosis detection

At harvest, cells were washed twice in PBS and detached from culture plates with a trypsin/EDTA solution. Next, live cells were incubated for 7 min in Hoechst33342 (10 mg/mL) at 37 °C and co-stained by propidium iodide (2.5 mg/mL). Samples were kept in the dark on ice for 15 min and then were immediately analyzed using a fluorescent Leica DMI6000 microscope and a 20× by dropping the samples on coverslips. At least 400 cells were used in the analysis. The ration of apoptotic and necrotic cells was counted as shown before^67^.

### Next-generation sequencing

Regions targeted by CRISPR/Cas9 and potential off-target regions were amplified using pairs of specific primers and Q5 polymerase. Amplicons were gel-purified and extracted using Qiagen gel extraction kit, quantified with a Qubit 2.0 Fluorometer (Life Technologies), and pooled in equimolar ratios. Adapters for Illumina sequencing were then attached. Libraries were sequenced with 250 paired-end reads using the MiSeq instrument (Illumina). FASTQC software and Geneious software were used for quality assessment, reference alignment, discarding low-quality reads/nucleotides, and calculating substitutions and indels. Per-base sequence quality and per-sequence quality scores in all groups were >30.

### Statistical analysis

Values were expressed as the mean ± standard deviation (SD) of triplicate experiments in SPSS software (SPSS 21.0.0.0). One-way ANOVA and Student’s t-test with Tukey’s HSD post hoc test were used to compare variables and calculate P-values to determine statistically significant difference in means.

## Supplementary information

Supplementary information
