# Supplementary material for: Suppressing the NHEJ pathway by DNA-PKcs inhibitor NU7026 prevents degradation of HBV cccDNA cleaved by CRISPR/Cas9
Source: Sci Rep. 2019 Feb 12;9:1847. doi: 10.1038/s41598-019-38526-6 (PMC6372644; doi:10.1038/s41598-019-38526-6)
Supplement: Supplementary file 1 — Supplementary information [file 41598_2019_38526_MOESM1_ESM.pdf]

Supplementary materials

Suppressing the NHEJ pathway by DNA-PKcs inhibitor NU7026 prevents degradation of HBV cccDNA cleaved by CRISPR/Cas9

Dmitry S. Kostyushev[1*], Anastasiya Kostyusheva[1], Sergey Brezgin[1,2], Dmitry Zarifyan[1], Anastasiya Utkina[1], Irina Goptar[1,3], Vladimir Chulanov[1,4]

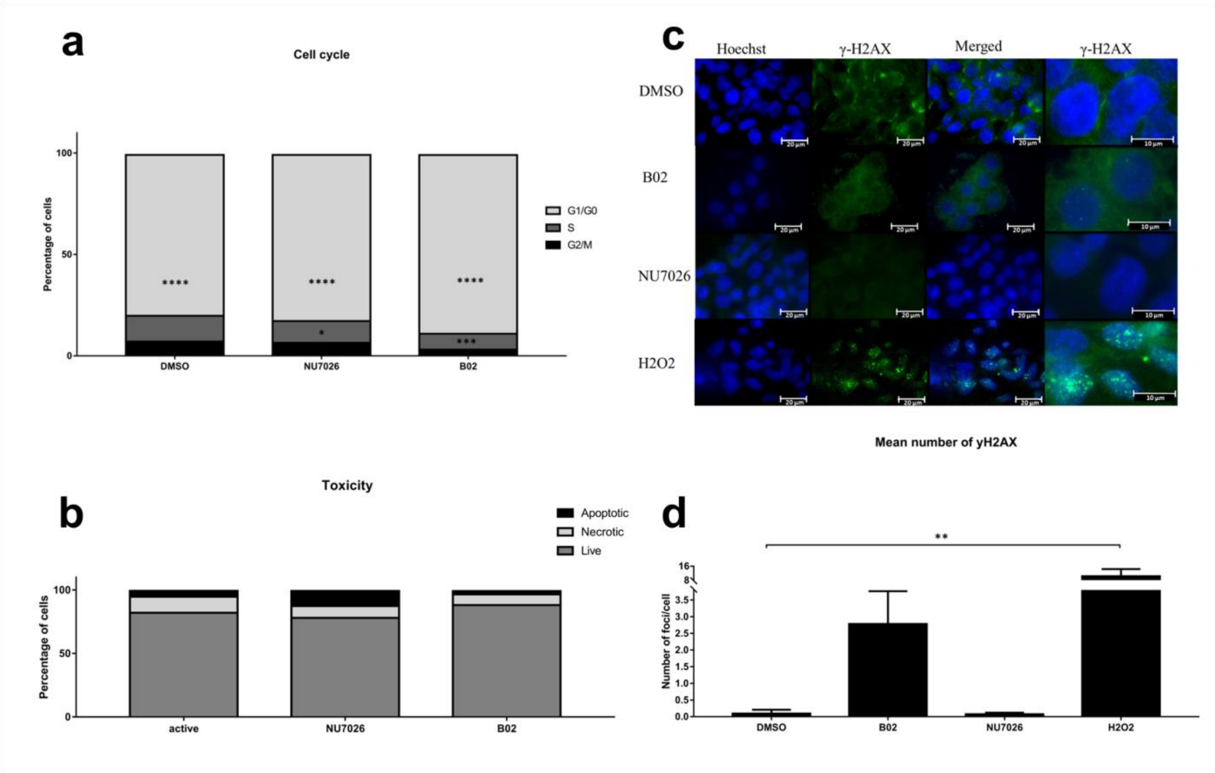

Figure S1. Toxicity of small molecules and their effects on cell cycle. (A) Effects of NU7026 and B02 on the cell cycle. HepG2-1.1merHBV cells were treated with the indicated small molecules for 24 h, detached by trypsin, and stained with the live dye DRAQ5. Cell cycle analysis was performed by flow cytometry. (B) Effects of NU7026 and B02 on the proportions of apoptotic, necrotic, and live cells. Cells were treated with NU7026, B02, or DMSO for 24 h, and then fixed and stained with propidium iodide and Hoechst33342. Proportions of apoptotic,

necrotic, and live cells in each group were calculated based on nuclei staining. For details, see Materials and Methods. (C, D) Genotoxicity of NU7026 and B02. HepG2-1.1merHBV cells were cultured with the indicated small molecules for 24 h, and fixed and stained with anti-yH2AX antibodies. Treatment with H_2O_2 for 1 h was used as the positive control. yH2AX foci were counted in a blinded manner. At least 100 cells were taken into analysis. Asterisks indicate statistically significant differences. *$p < 0.05$, **$p < 0.01$, ***$p < 0.001$, ****$p < 0.0001$.

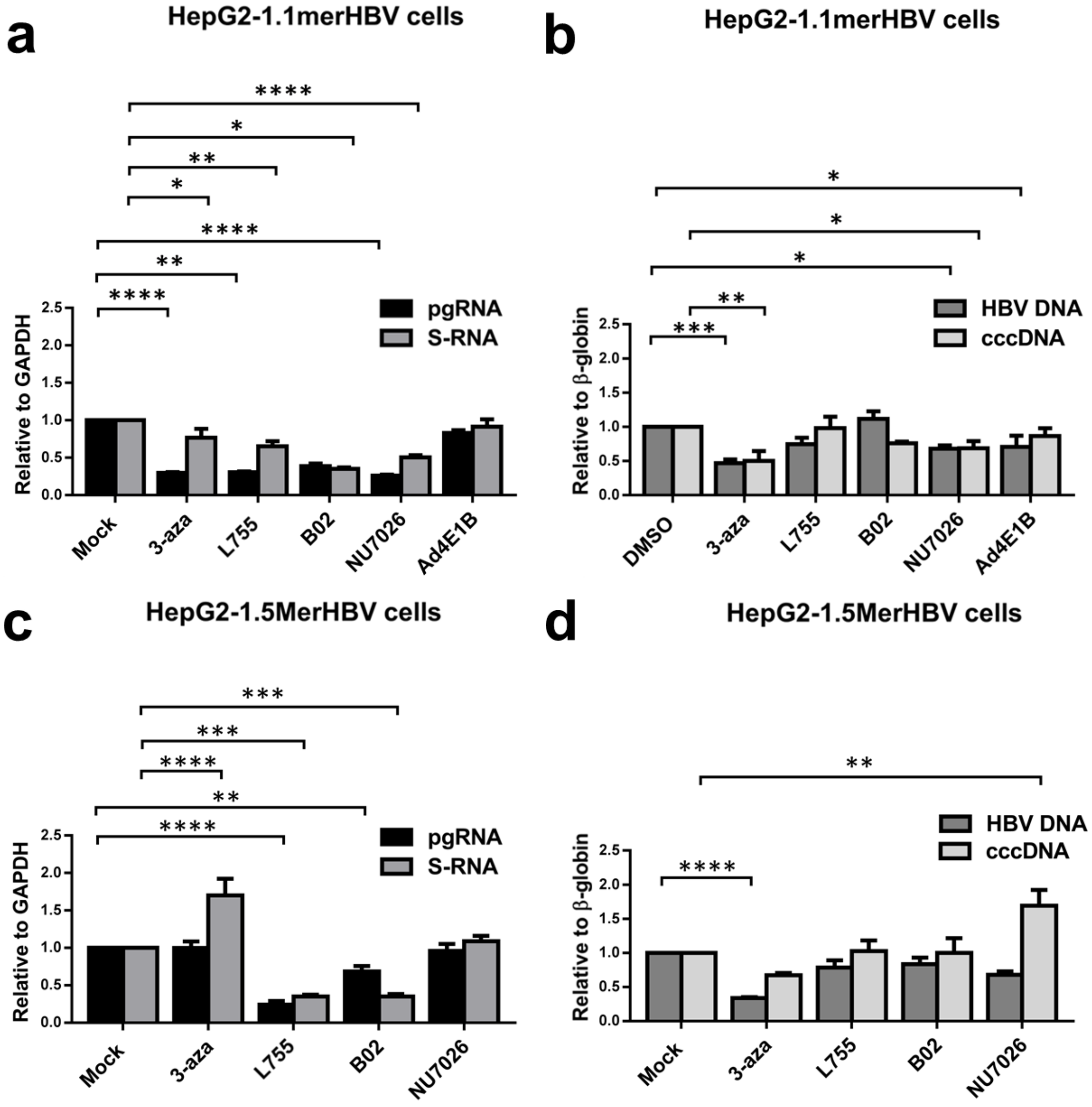

Figure S2. Effect of small molecules on the HBV life cycle. Alterations in HBV life cycle in HepG2-1.1merHBV (A, B) and HepG2-1.5merHBV (C, D) cells after 3 days of incubation with the indicated small molecules. Levels of pgRNA and S-RNA were measured by qRT-PCR with

TaqMan probes and normalized to GAPDH mRNA levels; HBV DNA and cccDNA levels were normalized to β-globin. Asterisks indicate statistically significant differences. *p < 0.05, **p < 0.01, ***p < 0.001, ****p < 0.0001.

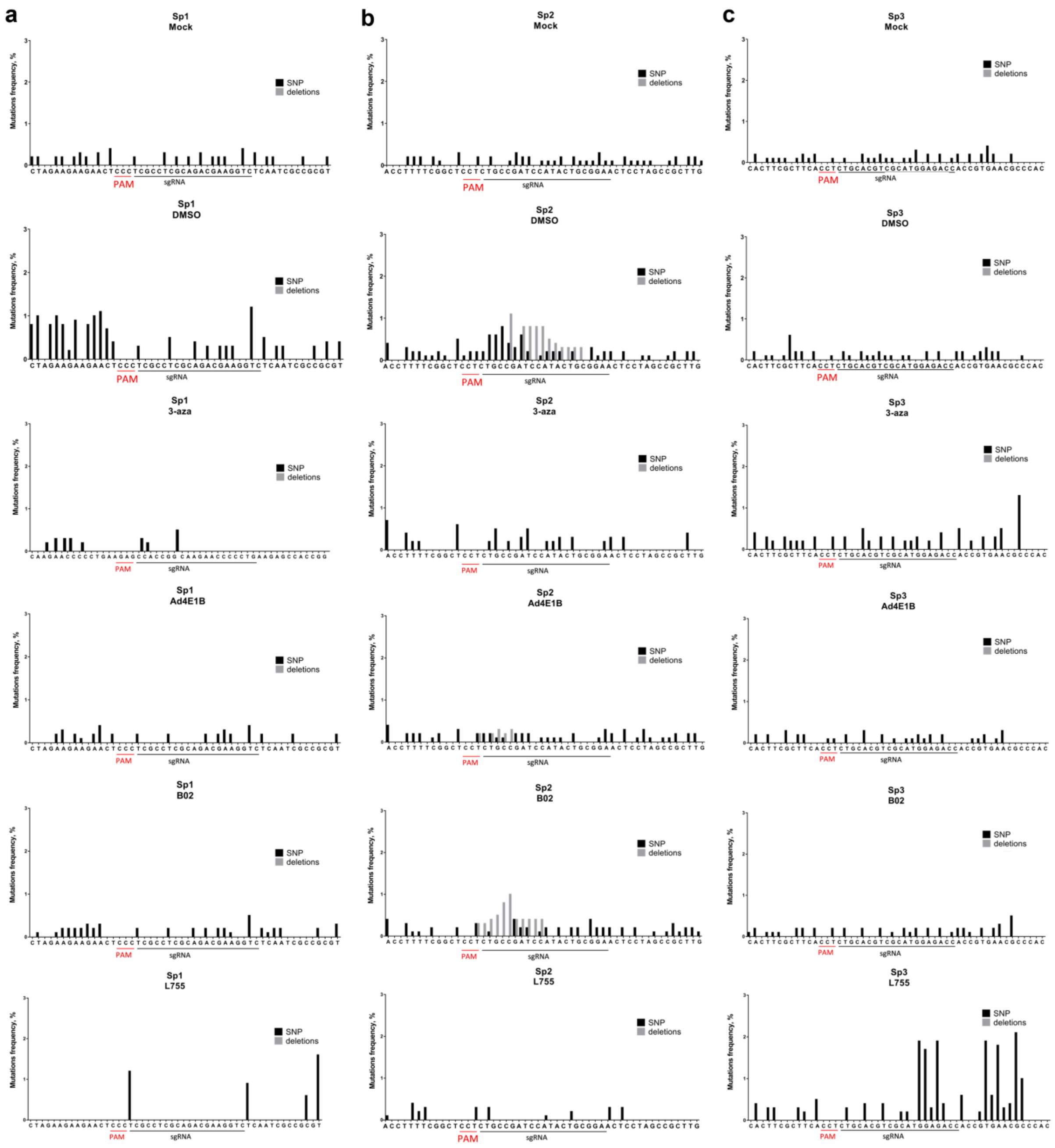

Figure S3. Typical insertion/deletion sequencing results. Snapshots of aligned sequencing data are presented for the mock-treated group, Sp2 + DMSO, and Sp2 + NU7026 groups. Treatment with NU7026 results in more complex mutations at CRISPR/Cas9 target sites.

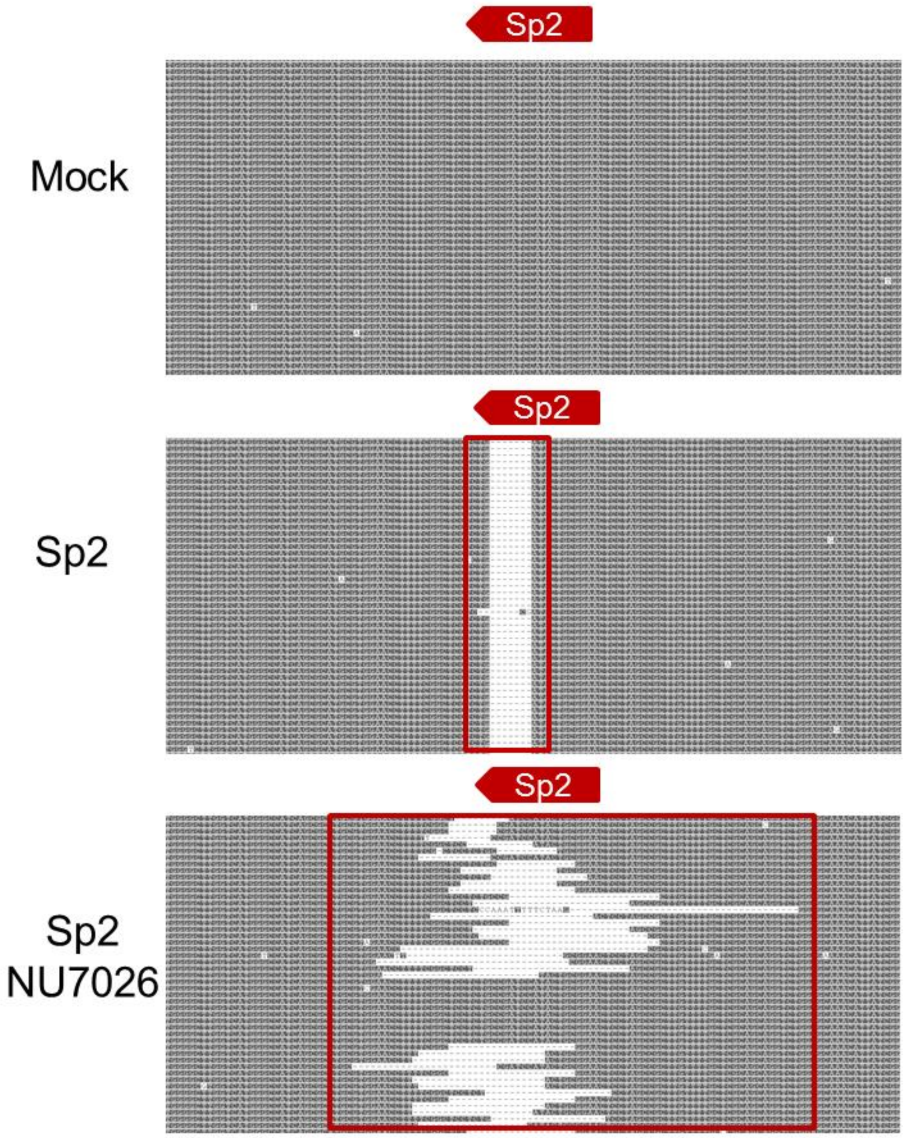

Figure S4. Deep sequencing of on-target sites. Cells were transfected with CRISPR/Cas9 and treated with a small molecule (3-aza, B02, or L755), or Ad4E1B protein was co-expressed. HBV cccDNA was isolated, and sites targeted by Sp1, Sp2, and Sp3 sgRNAs were sequenced by next-generation sequencing. Some indels were observed in the Sp2, Sp2 + Ad4E1B, and Sp2 + B02 treatment groups. Frequent mutagenesis was not observed in other groups.

Name	Sequence	Target
Sp1	GACCTTCGTCTGCGAGGCGA	Core/pre-core
Sp2	TCCGCAGTATGGATCGGCAG	EnhI
Sp3	GGTCTCCATGCGACGTGCAG	X-gene

Table S1. sgRNAs used in the study.

	Name	5'-3' nucleotide sequence	Melting T (°C)
1	ultramerSP1	TATATAGCTAGCAAAAAAAGCACCGACTCGG	See Table S3
2	ultramerU6_f	TATATAGGATCCGAGGGCCTATTTCCCATGATTCCTTCATATTTG	See Table S3
3	Sp1_f	GACCTTCGTCTGCGAGGCGAGTTTTAGAGCTAGAAATAG	See Table S3
4	Sp1_r	TCGCCTCGCAGACGAAGGTCCGGTGTTTCGTCCTTTC	See Table S3
5	Sp2_f	TCCGCAGTATGGATCGGCAGGTTTTAGAGCTAGAAATAG	See Table S3
6	Sp2_r	CTGCCGATCCATACTGCGGACGGTGTTTCGTCCTTTC	See Table S3
7	Sp3_f	GGTCTCCATGCGACGTGCAGGTTTTAGAGCTAGAAATAG	See Table S3
8	Sp3_r	CTGCACGTCGCATGGAGACCCGGTGTTTCGTCCTTTC	See Table

			S3
9	fGAPDH	CCAGGTGGTCTCCTCTGACTT	54
10	rGAPDH	GTTGCTGTAGCCAAATTCGTTGT	54
11	FA_Gapdh	FAM-AACAGCGACACCCACTCCTCCACC-BHQ1	54
12	cccDNA_f	CCGTGTGCACTTCGCTTCA	60
13	cccDNA_r	GCACAGCTTGGAGGCTTGA	60
14	cccDNA_probe	FAM-CATGGAGACCACCGTGAACGCCC-BHQ1	60
15	pgrna_f	GGTCCCCTAGAAGAAGAACTCCCT	62
16	pgrna_r	CATTGAGATTCCCGAGATTGAGAT	62
17	pgrna_probe	FAM-TCTCAATCGCCGCGTCGCAGA-BHQ1	62
18	srna_f	TCCTCCAACTTGTCCTGGTTATC	60
19	srna_r	AGATGAGGCATAGCAGCAGGAT	60
20	srna_probe	FAM-ATGATAAAACGCCGCAGACACATCCAGC-BHQ1	60
21	bglobin_f	V31-FEP-CE - AmpliSens® HPV HCR-Screen (CRIE)	60
22	bglobin_r	V31-FEP-CE - AmpliSens® HPV HCR-Screen (CRIE)	60
23	bglobin_probe	V31-FEP-CE - AmpliSens® HPV HCR-Screen (CRIE)	60

Table S2. Sequences of primers and probes used in the study.

1st PCR product

	Stock	Final	Per tube
MQ water			12.5 µL
Q5 buffer with 1.5 mM $MgCl_2$	5×	1×	4 µL

2nd PCR product

	Stock	Final	Per tube
MQ water			31.5 µL
Q5 Buffer	5×	1×	10 µL

dNTP mixture	5 mM each	0.2 mM each	2 µL
ultramerSP_r/ultramerU6_f	10 µM	0.25 µM	0.5 µL
sgRNA_f /sgRNA_r	10 µM	0.25 µM	0.5 µL
pLX-sgRNA		10 ng/ reaction	0.1 µL
Q5 Polymerase	5 U/µL	0.1 U/µL	0.25 µL
			20 µL

Cycling profile

95°C	2 min	
95°C	30 sec	
50°C	30 sec	×30 cycles
72°C	25 sec	
72°C	5 min	

dNTP mixture	5 mM each	0.2 mM each	5 µL
UltramerU6_f	10 µM	0.25 uM	0.5 µL
UltramerSP_r	10 µM	0.25 uM	0.5 µL
DNA templates of PCR reaction 1		10 ng/ reaction each	2 µL
Q5 Polymerase	5 U/µL	0.1 U/µ	0.5 µL
			50 µL

Cycling profile

95°C	2 min	
95°C	30 sec	
60°C	30 sec	×35 cycles
72°C	30 sec	
72°C	2 min	

Table S3. Protocols and cycling conditions for generating PCR products encoding sgRNAs.

		Mutations/1,000 bp			
sgRNA	Name	SNP	Deletions	Insertions	Total reads
Sp1	Mock	87,0	0,1	0,0	29,788
	DMSO	64,6	0,3	9,5	46,406
	3-aza	107,9	0,0	0,0	15,483
	L755	86,0	0,0	0,5	4,547
	B02	102,5	0,9	0,0	29,389
	NU7026	111,5	188,2	0,0	27,043
	Ad4E1B	91,2	0,0	3,4	28,008
Sp2	Mock	41,0	0,0	0,0	83,782
	DMSO	41,6	63,1	0,3	99,062
	3-aza	96,2	0,0	0,0	17,565
	L755	51,7	0,0	0,0	34,931
	B02	44,8	13,2	0,4	62,017
	NU7026	100,9	171,9	0,5	62,589
	Ad4E1B	48,4	9,2	0,0	49,170
Sp3	Mock	185,1	0,0	0,0	96,438
	DMSO	381,4	0,0	1,0	141,391
	3-aza	34,9	0,6	0,0	12,007
	L755	49,0	0,9	0,0	8,044
	B02	182,0	0,0	0,0	86,477
	NU7026	90,4	46,5	0,9	45,660
	Ad4E1B	61,5	0,9	0,0	49,305

Table S4. Deep sequencing of on-target regions in HBV cccDNA.
